# A Self-Reflection Program for Smoking Cessation in Adolescents: A Phenomenological Study

**DOI:** 10.3390/ijerph17031085

**Published:** 2020-02-08

**Authors:** Inok Sim, Eunjeong Hwang, Bora Sin

**Affiliations:** 1 Red Cross College of Nursing, Chung-Ang University, 84 Heukseok-ro, Dongjak-Gu, Seoul 156-756, Korea; bborasin@naver.com; 2 Sehan University, 1113 Samho-eup, Jeollanam-do, Yeongam-gun 58447, Korea; ejhwang@sehan.ac.kr

**Keywords:** self-reflection, adolescent, smoking cessation program

## Abstract

The study aimed to understand the experiences of adolescent smokers who participated in a self-reflection program for smoking cessation and to develop the theoretical basis for constructing similar programs. The program is unique from other smoking cessation programs in that it seeks to be creative and allow participants to establish an individualized vision for themselves. The participants, ten students from middle and high schools located in cities A and S, were interviewed right after the program ended. Data were collected from August to December 2019 and analyzed using a phenomenological approach to understand participant experiences in depth. The analysis revealed five major themes: ‘Uniqueness of the Program,’ ‘Perception of Smoking Cessation,’ ‘Positive Reflection on Life,’ ‘Understanding Others,’ and ‘A Search for Hope and Vision in Life.’ The findings revealed that their smoking behaviors were changed through self-reflection and enhancement of self-efficacy and that the program facilitated the formation of identity and vision for the future, which may indirectly strengthen the motivation for adolescent smokers to quit smoking. These findings suggest the need for a smoking cessation program that enhances self-concept and self-esteem. Moreover, it highlights the importance of follow-up research to ensure effectiveness and the need to develop programs with creative content.

## 1. Introduction

Adolescence is a transition period between childhood and adulthood that requires cognitive development, maturity, as well as adaptation to a variety of psychological and physical changes resulting from secondary sex characteristics [1].

One of the most challenging developmental tasks in adolescence is balancing dependence and independence within social relationships [2]. In fact, during adolescence, teenagers struggle to achieve independence from parents and tend to socialize with peers of both genders, which affect the formation of their self-concept and self-esteem [1]. Further, adolescents tend to emulate colleagues and friends in establishing standards of self-control and behavior [3,4].

According to the Korea Youth Risk Behavior Web-based Survey, the current smoking rate for middle and high school students is 6.7%, which shows a steady decrease since 2005, but has been stagnant since 2016 [5]. Similarly, the smoking rate of male students ranges from 6.3% to 6.7% since 2016 and that of females from 2.7% to 3.7%.

Smoking is known to have adverse effects on the body during adolescence, especially since the bodies are less developed than those of adults [6]. Further, smoking plays a role in the use of violence, sexual behavior, alcohol, and drugs as related to juvenile delinquency [7].

Previous studies report that the use of tobacco in adolescence is closely related to daily stress levels, family functioning, and self-efficacy [8,9]. On the other hand, economic factors show a significant increase in the frequency of smoking as students’ average monthly allowances increase [10].

To date, studies on factors related to youth smoking [4,11] and various programs, such as student counseling and health education to encourage smoking cessation and prevent secondhand smoke exposure, [12,13,14,15] have been developed. However, there is a lack of sequential intervention programs that aim to understand the factors related to teen tobacco use, provide in-depth and continuous management of teen smoking, and initiate a change in attitudes toward smoking by incorporating a meaningful vision into their lives. More importantly, it is time for continuous efforts to be developed and applied in the context of creative and practical programs.

Furthermore, it is necessary to develop a new program that enables young adults to envision themselves as future leaders of society, by fostering self-esteem, self-improvement, and positive thinking. The program content for adolescents is very crucial, since these adolescents are in a state of psychological transition, experiencing conflicts between self-consciousness and reality with many concerns about others’ expectations and their future. Considering these, this self-reflection program is specifically designed for adolescents, which can facilitate identity formation, a crucial developmental task for teenagers [16]. Moreover, this program incorporating interactive group activities such as choir performance may help form their identities through continuous identity exploration within supportive peer relationships [17].

Hence, in this study, the smoking cessation program focused on self-reflection and improvement. The aim of this study is to analyze the experiences of adolescent smokers, who participated in a holistic program that aimed to encourage quitting tobacco use and reflecting on oneself, using a phenomenological approach.

## 2. Materials and Methods

### 2.1. Design

Phenomenological research is a form of qualitative method that examines an individual’s subjective experiences within the world [18]. This methodology enables an understanding of rich data obtained from the lived experiences of the participants, and the goal is to clarify and reduce the meanings of the phenomenon from an individual’s lived experiences [18]. Therefore, this qualitative study adopted a phenomenological approach to gain a deeper understanding of the individual experiences of adolescent smokers who participated in the self-reflection and improvement program.

### 2.2. Participants

Participants of the study were 10 of the 33 students who voluntarily participated in the entire 1-4 stages of the program. The ten participants were interviewed twice, and if there were a need for additional information or clarification, they were set for a follow-up interview.

They P Middle School, P High School, and G High School located in G province, the Republic of Korea specifically in A and S cities. Both cities were areas with low socio-economic status, and in these three low-performing schools, there a large percentage of marginalized, poorly performing students who lacked social and emotional support from others such as parents and teachers. Likewise, many students in those schools gathered and engaged in deviant behavior such as violation of school rules and smoking. Accordingly, purposive sampling was done to obtain information-rich cases for the understanding of the experiences of a small number of carefully selected smoking adolescents, who were. low-achieving students engaged in delinquent behaviors and had been smokers of at least 6 months [19]. A summary of the general characteristics of the participants is listed in Table 1.

For ethical consideration, the study was conducted after receiving approval from the institutional review board at Chung-Ang University (1041078-201910-HRSB-290-01).

### 2.3. Program Design

Deci and Ryan’s (1985) Self-Determination Theory posits that there are two main types of motivation: intrinsic and extrinsic motivation [20]. Among these, intrinsic motivation, which is the natural and inherent drive to explore and pursue new possibilities associated with growth, can be effectively used to allow learning and positive development in adolescents [20,21]. To instill intrinsic motivation in students, they must attempt to reflect on themselves by recognizing the areas of consciousness and unconsciousness to discover their driving forces deeply rooted within themselves. Therefore, as a means of identifying internal motivations, the first stage ‘Self-Reflection Program’ and the second stage ‘Self-Improvement Program’ were organized for addressing intrinsic motivation.

The third stage, ‘Healing Concert Program’, was designed for fostering positive thinking and motivation and enhancing socialization skills. This is based on the findings by Hwang (2010) where positive attitudinal change and motivation enhancement towards quitting smoking was found as a result of smoking cessation program with musical therapy [22]. Additionally, Sim’s study (2016) stated that participating in a choir had given the participants an increased level of confidence and cooperation with others [23]. Further, Hanrahan (1999) proposed that self-reflection may enhance the level of intrinsic motivation as well as performance [24]. Hence, the program aims to provide an opportunity to reflect on themselves that can uplift the level of intrinsic and extrinsic motivation which are gained from choir performances and self-reflective activities.

Finally, the fourth stage ‘Vision Tour’ was organized for students to experience various environments and design their future with their own goals. Kuh (2009) stated that the campus environment, which includes buildings, people and everything physical on the campus, serves as a set of symbols larger than the individual items [25]. Additionally, Manning (2000) discussed that campus visitation includes various symbols that allow students to make their own meaning of their role as members of a college community [26]. Hence, visiting a college campus and government agency can help students to have goal-oriented attitudes in their lives by symbolic interaction with the environment.

The program was structured in a way to help students not only identify, assess and analyze their own physical, social, and psychological problems through self-reflection but also to understand themselves comprehensively and holistically in the context of living a healthy and happy life, which may all increase their motivation to quit smoking.

#### 2.3.1. Stage 1: Self-Reflection

The first stage of the program was conducted in a quiet space in each school for three hours per session, once a week for a total of four weeks. Details of the ‘Self-reflection’ stage are given below in Table 2.

#### 2.3.2. Stage 2: Self-Improvement

Participants in this stage are students from the first stage, and it was designed to identify factors and establish strategies for self-improvement after reflecting on oneself in a holistic and integrated manner. Additionally, it was planned to provide opportunities to familiarize themselves with each other in preparation for stage 3, the healing concert. The program was conducted over a period of 2 days and 1 night at a training facility located in G province. Details of the self-improvement stage are given below in Table 3.

#### 2.3.3. Stage 3: Healing Concert

The healing concert aimed to provide teen smokers with an opportunity to experience a sense of accomplishment through choir performances. Further, a choir, consisting of university student mentors, and dance and vocal ensemble groups composed of middle and high school students, also performed. The concert was designed to help participants develop social skills, self-confidence, and coping skills, to not only improve their ability to overcome all potential difficulties in their future but also to find the driving force for happiness in their lives.

After stage 2, the participants started their practices with school music teachers. The practice meetings took two hours once a week for a month and the students further practiced at their homes. The concert lasted two hours in a hall located in G province, and details of the healing concert are shown in Table 4.

#### 2.3.4. Stage 4: Vision Tour

Participants in this stage comprised of students who had completed all prior stages. The tour aimed to give these participants dreams and hopes for their future roles in the world, to facilitate future-oriented thinking and to instill hope that can help them establish specific directions for the future by visiting historical sites, institutions, and universities (Table 5).

### 2.4. Data Collection

The program intervention and data collection period were from August to December 2019, where the in-depth interviews with ten participants were done. Before starting the program, the basic demographic questionnaire was administrated to gain information such as gender, age, the educational stage when they started smoking, motives for smoking, daily cigarette consumption, and awareness of the health risks associated with smoking. After completion of the program, an interview meeting with the participants was scheduled by the school authorities. Moreover, to make the participants feel at ease, the meetings were held in spaces familiar to them such as school classrooms. A total of two in-depth interviews were conducted, and follow up interviews were made if there was a need to clarify or collect more data. The interview questions were about experiences before and after the program and changes in perception and thinking after the program ended. Further, the interviews were conducted 3 months after the program ended to find out whether positive changes were maintained. Researchers conducted the interviews until the point of data saturation, where new data tend to be redundant of the data previously obtained [27]. Accordingly, ten participants were interviewed.

Each in-depth interview took 30 minutes to an hour. Interview content was recorded based on prior consent, and additional details were written down by the researchers. During the first interview, participants were asked to express their expectations from the program in an open manner, and the second and third interviews were held to confirm the details provided in the first interview or to gain additional data. The interviews were unstructured containing open-ended questions such as ‘What did you experience in this program?’, ‘What did you learn about yourself through this program?’, ‘What did you experience regarding your plans for smoking cessation and the future?’ In addition, researchers made individual phone calls to further collect data and clarify areas of ambiguity when analyzing interview information.

### 2.5. Data Analysis

The phenomenological method aims to extract meaning from first-person accounts of experience and this study incorporated the phenomenological data analysis method of Colaizzi [28]. To ensure a general understanding of the data, the audio transcript was first thoroughly studied. The transcript was then reviewed to identify significant statements and their meaning, which were then categorized independently by two researchers. The categories were compared with each other and with the original transcript, and if there were any differences in the results, the researchers gave critical advice to each other and were resolved through discussions. The categories were then grouped under overarching themes, and the researchers analyzed theoretical statements based on the data collected, which the researchers analyzed to formulate a structured view. Lastly, the researchers verified the findings with the participants to confirm that the results were consistent with their intended meanings.

## 3. Results

The analysis of smoking adolescents’ experiences with the self-reflective smoking cessation program is shown in Table 6. The analysis of the original data revealed 60 meaning units, out of which overlapping contents were merged into 26 formulated meanings. These were then classified into seven categories and five themes. The five themes are shown in Table 6 as ‘Uniqueness of the program,’ ‘Perception of smoking cessation,’ ‘Positive reflection of life,’ ‘Understanding others,’ and ‘A search for hope and vision in life.’

### 3.1. Uniqueness of the Program

Participants felt that this particular smoking cessation program was different from other smoking cessation programs that they had attended because other programs were boring, unoriginal, and lacked complex intervention. They also explained that their level of participation and expectations from the program increased as the program progressed. Positive effects arose by repeatedly meeting new mentors and participants, which allowed their minds to heal and to develop an interest in things and pursuits other than just smoking.

*“I thought it would be boring and unoriginal again. I felt the kind mentors treated and respected me as an ordinary student. I felt close to them and it made me want to take the program again. I thought it was getting better and better expecting what to do next.”*

*(Participant H)*

*“It was good to have a chance to meet new friends and talk about different topics. We traveled together, rehearsed at concerts, made a stage together and became close.”*

*(Participants A and J)*

*“My parents nagged me everyday about quitting smoking. I wanted to take a shot as I thought it would be easier to meet new friends and do it as a group than to do it alone. It was a special program as I reflected on my thoughts and attitudes.”*

*(Participants B and D)*

### 3.2. Perception of Smoking Cessation

Participants revealed that they had received several educational lessons on the harmful effects of smoking since grade school, but they still had questions as to why they should quit smoking. Further, they did not take the negative effects of smoking seriously since they had not experienced any of these effects directly yet. However, they explained that they discovered the reasons why they started smoking by reflecting on their youth and attitudes towards life. Additionally, they shared that they realized they behaved and thought differently as compared to others.

“Self-reflection has allowed me to think about how I behaved and grew up as a child. After I entered middle school, I just started smoking with my friends without much thinking... I think that has become a habit. And I thought that people would think someone who smokes is a bad kid…. As a result, I drifted apart from good friends who also study well. I think I just lived like this.”

*(Participant C)*

“My grandfather still smokes, and he’s healthy. The last time I took part in non-smoking education, they just scared me with lung cancer and stuff. There were no attempts to make me realize and understand by myself to quit smoking, so I started not liking such educational programs….”

*(Participant E)*

Moreover, over the course of the program, the participants realized that their need to smoke was unnecessary and they recognized the consequences of smoking.

“I do not know exactly why I should quit smoking… but I did not have any health problems from smoking. People just said that it is bad, but they did not acknowledge and respect me… The program didn’t just educate me that smoking was bad, but it made me face the consequences of smoking behavior to realize the seriousness of the problem. Also, teachers did not treat me like a bad kid. They respected me all throughout the program….”

*(Participant I)*

### 3.3. Positive Reflection of Life

Participants expressed remorse for their past behavior. Some of the participants regretted having started smoking so young and not being aware of the seriousness attached to smoking. Some of them even thought that they were worthless and helpless and felt sad about not having any dreams or goals.

“Self-rationalization? Yes, it is. Until now I thought that smoking may be harmful but not painful. What can I do about it? Smoking is none of other’s business, but now I realize I was wrong.”

*(Participant F)*

“I think I lacked confidence. I felt helpless and I compared myself with others, so I believe I started smoking to look confident. I should not have done that. But through the program, I realized that there were many things that are worth giving a shot.”

*(Participant E)*

“I didn’t have a dream. I didn’t even know why I should have a dream…. I believe that if I try hard enough, I can become anything—even a congressman.”

*(Participant C)*

### 3.4. Understanding Others

Participants shared that they felt apologetic and realized the value of their families as a result of the self-reflective smoking cessation program. They were previously not aware that smoking could affect their families’ health since they were ignorant of the people around them. Additionally, they realized the importance of their family and themselves. Although they had not been aware of whether smoking could affect their families’ health because they only cared about themselves and were not interested in the people around them, they began to experience the negative consequences of smoking as they reflected on their lives and became aware of their behavior during the program. Moreover, participants elucidated that they understood their parents’ feelings and were genuinely apologetic for having smoked during adolescence. As such, they mentioned that they valued the experience of realizing how their actions affect their families as well as themselves.

“I was nervous but proud of myself when we performed during the Healing Concert what we practiced dancing and singing… Because the program was interesting, it was easier to get closer to each other. Our attention was diverted from smoking, so we forgot to smoke….”

*(Participant A)*

“I think I only thought about myself. I think that’s why I have been showing problem behaviors by smoking intentionally. But I think it could have been a burden to my mom and dad. My mom must have been very tired. It also smells, yet my mom doesn’t even smoke…… I feel very sorry for my mom….”

*(Participant A)*

“I really thought that I would get hit when my parents found out that I was smoking? But they were gentle with me. At that time, I just liked the fact that I was not being scolded, but now when I think about it, they were trying to be patient even if they were having difficult times with me. Even though they were very upset….”

*(Participant E)*

Participants also experienced changes in understanding their friends by wondering about the motives of their smoking, and elucidating how they were similar and different from their friends. Moreover, the statement, *“I won’t recommend a cigarette to a friend,”* suggests that they placed importance on the meaning of friendship.

“Sometimes I feel like I don’t want to do anything, and it is annoying when someone says something, but so did my friends.”

*(Participant A)*

“I now realized during the program that my thoughts can be different from others, and everything goes fine if we understand this.”

*(Participant D)*

### 3.5. Searching for Hope and Vision in Life

In addition to regretting their past, participants also formed expectations regarding their future. They thought there was no need to smoke after experiencing increased self-esteem. Although they did not come up with a definitive dream or plan for the future, they felt hopeful in having loose ideas. As such, most students became optimistic about being a new person with a set of expectations and hopes for the future.

“Although I do not have an absolute dream, Until now, school teachers kicked me out of the classroom, saying my actions were wrong. Anyways, getting to have a dream feels good, and I hope to be so.”

*(Participant I)*

“I started to think, If my self-esteem increases, then I will not have to smoke any longer.” Coming across chances, just like this program, to see others live happily, gave me hope, and college tours made me think, ”Would I be able to go to that college if I study hard enough?”

*(Participant E)*

“My mentor asked me to think deeply about what I can do best. Since I was younger, I loved sports and do well at it? So I decided to work harder and have sports as my specialty and become someone better.”

*(Participant G)*

“From now on, I have set new goals for me. I will quit smoking and become a new person!”

*(Participant B)*

## 4. Discussion

The study examined the experiences of teen smokers who participated in the smoking cessation program based on self-reflection. The results of this study discussed the participants’ experiences with the program, strengths, and limitations according to five commonly analyzed themes.

Firstly, adolescent smokers explained that the self-reflection program had a unique and creative approach unlike other programs, which developed their interests and increased their expectations. Participants shared that other programs they had attended merely focused on the dangers of smoking, such as lung cancer, which only created fear among the participants. Moreover, they developed a sense of intimacy and stability in relationships by experiencing changes in the ways of understanding and perceiving others through various group activities in every stage such as chorus practices. These findings are consistent with Flannery and Smith’s study in which education and positive emotions were improved when an environment creates intimacy and trust within social relationships [29]. These positive results have been shown to develop intrinsic motivation for participants to make their own decisions and to feel confident regarding their actions. Additionally, Kiemer, Gröschner, Kunter and Seidel reported that when students experience the teachers as autonomous and competence-supportive, they were more likely to experience intrinsic learning motivation [30]. Therefore, it is essential to develop creative content that is specific for teenagers and designed to arouse curiosity, which can then increase participation and satisfaction of participants. Furthermore, it is crucial for the program hosts’ attitude to demonstrate that everyone, especially low-performing students, is worthy of respect.

Secondly, the participants had an opportunity to contemplate and be aware of their smoking behavior. According to the data from the basic demographic questionnaire, before attending the program, they were vaguely aware of the problems of smoking but never had a chance to associate it with their life. However, they could identify the causes of their smoking and related problems through the program by reflecting on their experiences and exchanging ideas with each other about tobacco use. It was also noted that the motives for smoking, as perceived by the participants, were often a friend’s suggestion to smoke, low academic achievement, bullying, a sense of alienation, the stress in everyday life, or an urge for novel experiences, among others. This parallels with the result that most teenagers are more likely to smoke when there is a negative influence from others, interpersonal problems, uneasy mental health, and poor academic achievement [31].

Teen smokers lack the opportunity to explore themselves in-depth, reflect on their delinquent behaviors, and discover life values. For these adolescents, it is relevant not to take a one-sided approach to warn them about the consequences of smoking or the need to quit smoking, but to strengthen their strengths and positive thinking. This is supported by Adams and Oliver’s (2019) findings which established that one’s identity and socializing in groups allowed adolescents to recognize their problems and explore strategic ways to manage them [32].

Third, participants were able to reflect on their lives while participating in the program. Self-reflection is a process of exploring one’s own experiences, which yields new understandings from different life experiences [33]. Meanwhile, one of the monumental developmental tasks that individuals face during adolescence is identity formation. Accordingly, teenagers continue to realize who they are and understand their tendencies. Some participants in this study also realized through self-reflection that they had been living without hopes and dreams, and with negative thoughts and low self-esteem.

Zelazo and Doebel posit that when an adolescent is self-conscious and has control over him/herself, he/she can establish changes in thinking and behavior, and pursue personal goals and responsibility, thereby predicting healthy adaptation [34]. As such, there is a need to create an environment where teenagers can have a broader perspective and increase their abilities. Future programs for adolescents should be developed with continued interest in giving them opportunities to promote self-regulation. 

Fourth, the participants felt apologetic toward their families and realized how precious their family was to them. Additionally, they became aware of the differences and similarities between themselves and their friends. Family and friends, who are the closest people in a teenager’s daily life, have a tremendous influence on them. Notably, some of the factors that contribute to smoking among adolescents are negative consequences in relationships within families or pressure and recommendations from friends to smoke [35]. The participants’ experiences of self-reflection seem to have broadened the way they understand others by allowing them to recognize how they are affecting the people around them and to develop harmonious relationships with colleagues [36]. In addition to giving up smoking for family members through the self-reflection program, it was found that the participants encouraged their friends not to have bad habits, such as using tobacco and are less likely to participate in smoking themselves.

Fifth, some participants were able to envision their future life through reflection, discover their strengths, and become aware of their potential. Such attention and reinforcement of abilities bolstered their self-efficacy, which changed their attitudes towards smoking. Further, confidence in themselves and in their ability to change their behavior may have motivated them to quit smoking. A study by Ayar et al. also explains that self-efficacy and smoking are negatively correlated, which further supports the assumption of this study that an increase in self-efficacy motivates smokers to stop smoking [9].

## 5. Conclusions

This study was conducted using the phenomenological method in order to gain a deeper understanding of the experiences of adolescents who completed a self-reflective smoking cessation program and to provide a theoretical basis for the designing of future anti-smoking programs.

The five common themes experienced by the participants included the ‘Uniqueness of the program,’ ‘Perception of smoking cessation,’ ‘Positive reflection of life,’ ‘Understanding others,’ and ‘A search for hope and vision in life.’ The smoking cessation program in this study is different from other anti-smoking programs in that it seeks to be creative and allow participants to establish an individualized vision for themselves. Such goals of the program were based on facilitating self-reflection throughout the four gradual phases of the program regarding participants’ goals and hopes, as well as the maintaining of social relationships with instructors and colleagues. The program is also unique in that smoking behavior was changed through self-reflection and enhancement of self-efficacy, which is one of the positive factors in the crucial developmental task of self-identity formation.

Through this program, the participants gained a wider understanding of others through self-reflection and found their happiness and vision in life. This experience may indirectly suggest that quitting smoking makes people feel positive about life and creates new perspectives. The researchers hope that the content discussed in this study could be used as fundamental data in the development of anti-smoking programs for adolescents in the future.

Based on the discussion and conclusion of this study, the following limitations and suggestions are to be considered in future studies. First, it is limited to establish a concrete idea using the derived concepts from this study as there are limitations in gender and age, as the research was conducted with a small number of participants. Second, recognizing the need for further systematic and practical research based on the results of this study, the researchers would suggest that studies be conducted with a randomized and larger number of samples and that the data from closed-ended questionnaires be integrated into the analysis to increase the credibility of the study. Lastly, the researchers would suggest that there is a need to utilize creative education programs to enhance self-efficacy and self-reflection.

## Figures and Tables

**Table 1:** General Characteristics.

Characteristics		Mean ± Standard Deviation or *n* (%)
Gender	Male	10 (100%)
Age		16.1 ± 1.10
Educational stage at starting smoking	Middle School	6 (60%)
	High School	4 (40%)
Smoking motives	Stress	1 (10%)
	Curiosity	4 (40%)
	Friend’s suggestion to smoke	5 (50%)
Daily cigarette consumption	Less than half a pack of cigarettes	7 (70%)
	A pack of cigarettes	3 (30%)

**Table 2:** Details of the Self-Reflection stage.

Stage	Number of Participants	Frequency	Program Content	
Self-reflection	32 participants	Once a week for a total of 4 weeks	Week 1	- A holistic and comprehensive understanding of oneself
				- Finding oneself through meditation
			Week 2	- Art therapy with experts to discover their one’s inner world
			Week 3	- Self-reflection and changing one’s mind through discussion with mentors
			Week 4	- Establishing a positive perception of “personal boundaries” and expanding one’s living space
				- Writing an autobiography using pictures from the past to make them realize how important they are
				- Making plans for one’s future

**Table 3:** Details of the Self-Improvement stage.

Stage	Number of Participants	Duration	Program Content	
Self-improvement	32 participants	Two days and one night	Expert seminar	- Lecture on the meaning of life for adolescents
				- Strategies for self-improvement
			With mentors	- Sharing of experiences in quitting smoking with mentors
			With group members	- Self-exploration, self-reflection and changing one’s mind

**Table 4:** Details of the Healing Concert.

Stage	Number of Participants	Program Content
Healing Concert	32 participants	- Developing one’s potential and enhancing the abilities of low-performing students through choir activity
		- Self-development through practicing chorus
		- Interaction with friends through music and Maintenance of social relationships
		- Achieving healing and confidence through chorus singing
		- Collaborating and building trust with musical instructors and colleagues
		- Formation of self-esteem, and creating a sense of accomplishment as well as future challenges through participation in the concert

**Table 5:** Details of the Vision Tour.

Stage	Number of Participants	Duration	Program Contents
Vision Tour	32 participants	A full day group tour (visits to university and government agency)	- Setting a direction for the future through a vision tour
			- University campus tour to experience the actual learning environment
			- College laboratory tour where one can have hands-on experience with a detailed selection of majors
			- A tour of major institutions that addressed future-oriented life (the National Assembly, the National Museum and the National Library)

**Table 6:** Analysis of experiences of the Smoking Cessation program.

Theme	Category	Formulated Meaning
Uniqueness of the program	Expectations arose from the differences with other programs	· Getting away from the idea that all smoking cessation programs are boring and unoriginal
		· Curiosity arose from the differences with other programs that promoted rote learning
		· Waiting to participate in the program and raising expectations from the program
		· Healing the mind with the help of mentors and colleagues
		· Opportunity to get intimate with diverse types of people
		· Expecting a program that can change one’s smoking habits
Perception of smoking cessation	Finding the causes of their smoking behavior	· Looking back on one’s youth and attitudes towards life
		· Becoming aware of the cause and reason for smoking
		· Thinking about others’ behavior and thoughts
	Recognizing that it is unnecessary to smoke	· Feeling unnecessary and insensitive towards smoking
		· Recognizing the severity of smoking
Positive reflection of life	Regret and reflect on one’s life	· Recognizing the health risks of tobacco smoking
		· Feeling regret for having started smoking too early
		· Thinking of how one’s self-esteem is low
		· Feeling a lack of dreams and goals
		· Reflecting on negative attitudes
Understanding others	Understanding family	· Recognizing that smoking also affects a family’s health
		· Becoming aware of parents’ feelings
		· Feeling sorry for parents
		· Acknowledging the importance of family
	Understanding friends	· Knowing why friends smoke
		· Recognizing the similarities and differences between friends and oneself
		· Feeling that one should not ask a friend to smoke
A search for hope and vision in life	Recognizing one’s strengths and committing to life goals	· Recognizing that there are more worthy pursuits than smoking
		· Engaging the need to boost one’s self-esteem
		· Becoming curious about one’s goal in life and the future
